# Mechanisms of Aerobic Exercise Impairment in Diabetes: A Narrative Review

**DOI:** 10.3389/fendo.2018.00181

**Published:** 2018-04-18

**Authors:** Matthew P. Wahl, Rebecca L. Scalzo, Judith G. Regensteiner, Jane E. B. Reusch

**Affiliations:** ^1^Division of Endocrinology, University of Colorado School of Medicine, Aurora, CO, United States; ^2^Veterans Administration Eastern Colorado Health Care System, Denver, CO, United States; ^3^Center for Women’s Health Research, Department of Medicine, University of Colorado School of Medicine, Aurora, CO, United States; ^4^Division of General Internal Medicine, University of Colorado School of Medicine, Aurora, CO, United States

**Keywords:** insulin resistance, mitochondrial dysfunction, nitric oxide synthase, microvascular, muscle perfusion

## Abstract

The prevalence of diabetes in the United States and globally has been rapidly increasing over the last several decades. There are now estimated to be 30.3 million people in the United States and 422 million people worldwide with diabetes. Diabetes is associated with a greatly increased risk of cardiovascular mortality, which is the leading cause of death in adults with diabetes. While exercise training is a cornerstone of diabetes treatment, people with diabetes have well-described aerobic exercise impairments that may create an additional diabetes-specific barrier to adding regular exercise to their lifestyle. Physiologic mechanisms linked to exercise impairment in diabetes include insulin resistance, cardiac abnormalities, mitochondrial function, and the ability of the body to supply oxygen. In this paper, we highlight the abnormalities of exercise in type 2 diabetes as well as potential therapeutic approaches.

## Scope

The prevalence of diabetes is rising rapidly in the United States, with a 75% increase from 1988 to 2010 (1). There are now 30.3 million people in the United States with diabetes (2). Diabetes is associated with a greatly increased risk of cardiovascular mortality, which is the leading cause of death in adults with diabetes (2–4). In 2015, diabetes was the seventh leading cause of death in the United States (2). Total direct and indirect costs associated with diabetes globally may have been as high as 1.3 trillion USD and are expected to rise (5, 6).

Regular exercise is a cornerstone of treatment for diabetes. In the 2018 Diabetes Standards of Care publication, the American Diabetes Association recommends most adults with type 1 and type 2 diabetes (T2D) should engage in 150 min or more of moderate to vigorous intensity physical activity plus two bouts of resistance exercise per week (7). Despite the well-established health benefits of exercise, paradoxically only 51% of adults in the USA meet the recommended aerobic exercise guidelines (8). Exercise training is associated with lowering blood pressure, improving insulin sensitivity, and glucose control, improving lipoprotein profile and playing an important role in weight management among other benefits (9). More recent evidence shows that moderate to high volumes of aerobic activity are associated with significantly lower cardiovascular and overall mortality risks in both type 1 diabetes and T2D (10). In contrast, physical inactivity or sedentarism, is known to have deleterious health effects in people with diabetes (11, 12).

The focus of this review is to outline recent advances in understanding the interaction between diabetes and impairments in aerobic exercise function. There are numerous other chronic conditions that are known to impact exercise capacity including smoking (13), obesity (14, 15), and hypertension (16–18). The mechanisms underlying these impairments may overlap with or differ from those leading to impairments in people with diabetes. Past studies from our group have addressed this potential overlap by controlling for BMI, weight, activity level, and excluding potential participants with hypertension, chronic kidney disease, or smokers (19).

## Clinical Impact

People with diabetes have physiologic exercise limitations and decreased cardiorespiratory fitness (CRF). More specifically, people with diabetes have approximately 20% lower maximal oxygen uptake (VO_2_ max) when compared with those without diabetes (20–25). This is important because reduced VO_2_ max is linked to increased cardiovascular mortality (11, 20, 22, 26–28). A second exercise limitation is that individuals with T2D have slower oxygen uptake kinetics with constant-load exercise (21, 29), indicating decreased ability to adapt to an acute change in the demand for oxygenation at the beginning of exercise. Our group has reported that, perhaps related to the aforementioned physiologic limitations, people with diabetes report greater perceived exertion compared with their non-diabetic counterparts; that is, exercise seems more challenging to them, even at very low-work rates (30).

We and others have characterized many individual impairments in key cardiac and vascular measures associated with CRF impairment in T2D including insulin resistance, endothelial dysfunction, decreased myocardial perfusion with exercise, abnormally increased pulmonary capillary wedge pressure (PCWP), decreased limb blood flow, and skeletal muscle mitochondrial dysfunction (20–24, 29, 31–36). CRF impairment in T2D is present in youth as well as adults, even those with recent onset diabetes and no clinically apparent cardiovascular disease (23, 24). The CRF impairment appears to be independent from effects of obesity or decreased habitual physical activity (19–24, 37–41). Due to methodologic limitations to date, we lack critical knowledge about how each of the associated abnormalities of CRF mechanistically contributes to the CRF impairments. In addition, we lack information about which of the abnormalities are potentially reversible vs non-reversible. The goal of this review is to discuss underlying mechanisms of decreased CRF in T2D and to highlight newer data suggesting a microvascular contribution to the impaired exercise capacity associated with T2D.

## Exercise as Intervention

Given the overwhelming importance CRF plays in health and the importance of exercise training in diabetes management, it is critical to characterize diabetes-related physiologic exercise limitations in order to identify therapeutic strategies to improve CRF. Physical activity has been seen to prevent T2D even in the absence of weight loss (42). Multiple studies have documented that exercise training generally improves exercise function in T2D (20–22, 29, 43–49). It is less clear if a clinical intervention with exercise training reduces mortality in established T2D. For example, the Look Ahead trial did not find a survival advantage associated with lifestyle/exercise intervention (50). In this study, there was a very low-event rate (due in part to the overall decline in CV mortality in people with DM likely related to the use of improved CV risk factor modification over the time period of the study). There were, however, other benefits resulting from lifestyle modification in the Look Ahead Study including better quality of life, lower medical costs, and reduced need of diabetes medications in the intensive lifestyle intervention group (51). We have previously shown that, despite improvement in CRF after formal exercise training, exercise impairments persist in people with T2D relative to similarly trained people without T2D, possibly suggesting a mixture of modifiable and fixed defects (Table 1) (21).

**Table 1:** Decreased exercise capacity in type 2 diabetes (T2D)—exercise training intervention.

	Lean control subjects	Overweight control subjects	T2D subjects
*N*	10	9	8
Age (years)	37 ± 6	37 ± 6	43 ± 7
Fasting glucose (mmol/l)	4.89 ± 0.43	5.12 ± 0.67	11.90 ± 3.80*
HbA_1c_ (%)	6.3 ± 2.8	5.4 ± 0.5	9.5 ± 1.9*
VO_2_ max (ml/kg/min)			
Before	25.1 ± 4.7	21.8 ± 2.9	17.7 ± 4.0*
After	26.0 ± 6.0	23.0 ± 1.8^†^	22.4 ± 5.5*^†^
RER			
Before	1.13 ± 0.08	1.12 ± 0.06	1.16 ± 0.13
After	1.12 ± 0.13	1.15 ± 0.05	1.12 ± 0.03
Heart rate (bpm)			
Before	174 ± 15	167 ± 12	166 ± 11
After	167 ± 12	164 ± 10	164 ± 18

*This table is adapted from Brandenburg et al. (21). Copyright 1999 by the American Diabetes Association. These data demonstrates reduced VO_2_ max in T2D compared with lean control and overweight control subjects. Note there is no difference in baseline physical activity (assessed by low-level physical activity recall) across all groups*.

*Data are mean ± SD*.

*P < 0.05 for difference between the group with diabetes and the other two groups.

*^†^P < 0.05 for difference between before and after exercise training*.

## Assessment of Potential Mechanistic Causes of Exercise Impairment in T2D

Previous research from our laboratory has demonstrated a significant positive correlation between insulin sensitivity and VO_2_ max (20, 31). As proof of concept, we elected to examine whether an insulin sensitizer has the capacity to improve exercise function. Our group investigated the effects of rosiglitazone, a thiazolidinedione (TZD), on exercise capacity in individuals with diabetes (31). TZDs augment insulin responsiveness of the skeletal muscle and adipose tissue. This study demonstrated significantly improved VO_2_ max (7% increase) in participants with diabetes that were treated for 4 months with rosiglitazone 4 mg/day (31). Insulin sensitivity, measured by HOMA, and endothelial function, measured by ultrasound of the brachial artery, were also significantly improved in the rosiglitazone-treated group. Since multiple physiological factors correlated with improvement in CRF, the relative contributions to improved exercise capacity from changes in insulin action and improvements in vascular function with TZD remain unclear.

Our findings with rosiglitazone and CRF were reproduced by Kadoglou et al. (52). In addition, they demonstrated an added benefit of exercise training plus the TZD (52), a finding which was recently substantiated in an animal model (53). The impact of TZD on exercise capacity appears to differ depending upon disease status, as people with T2D and cardiovascular disease did not improve VO_2_ max (54). Taken together, these publications support the proof of concept that targeting insulin action in diabetes is associated with improvement in VO_2_ max in uncomplicated T2D. Rosiglitazone is no longer commonly used in diabetes treatment, due in part to concerns that it increased risk of cardiac events [a claim the FDA later disputed (55)], and thus further research with other insulin sensitizers may be warranted.

Metformin, a known stimulator of AMP-activated protein kinase, has also been evaluated for its effects on CRF. A study published in 2008 by Braun et al. investigated the effects of metformin on exercise capacity in healthy individuals and noted metformin decreased VO_2_ max by 2.7% when compared with controls (56). A similar study in people with insulin resistance by HOMA IR also demonstrated a significant decrease in VO_2_ peak with metformin for 3 months (57). However, evaluation of the impact of metformin on VO_2_ peak has not been determined in people with diabetes. Limited investigations have addressed the response to metformin in people with metabolic disease and the data are mixed regarding CRF and metformin. A recent study showed a significant decrease in VO_2_ max in people with metabolic syndrome following 6 weeks of metformin administration (58). However, previous investigations in people with prediabetes demonstrated no effect of metformin on CRF when administered without a concomitant exercise intervention and no metformin-associated impairment of the response to an exercise training intervention (59, 60). Metformin is reported to inhibit complex 1 of the mitochondrial respiratory chain (61, 62). It has been postulated that this action of metformin may be responsible for the observed reduced CRF in people with diabetes. As metformin is first-line therapy for the treatment of diabetes, further studies are necessary to determine the impact of this widely used pharmacological agent on CRF in the primary population where it is prescribed.

Epigenetic modifications have been noted in T2D (63) and research also shows that acute exercise results in DNA hypomethylation in promoter regions in human skeletal muscle (64). There are suggestions in the literature that a physiological challenge, such as diabetes, may change these acute epigenetic responses (65). This is an area of intensive investigation under the NIH Common Fund Molecular Transducers of Physical Activity Program. Once the changes in the healthy population are understood, we will be in a better place to understand whether there are differences in the adaptive response in the context of diabetes.

## Mitochondrial Dysfunction

Diabetes is associated with mitochondrial dysfunction in cardiac tissue and skeletal muscle (66, 67). In addition to other muscle tissue, we reported that DM affects mitochondrial function in the vasculature (68). This finding is pertinent because mitochondria, when dysfunctional, can decrease normal vasomotion and generate excess vascular reactive oxygen species (ROS) (69, 70). Vascular ROS are related to vascular inflammation and vascular stiffness—precursors of clinical cardiovascular disease (71). Prior studies in healthy subjects demonstrate that exercise improves skeletal muscle and vascular mitochondrial function and decreases mitochondrial damage (72–76). However, our group found that rats with hypertension or metabolic syndrome and mild diabetes did *not* have the mitochondrial improvement in the aorta that was demonstrated in control rats after exercise training (68). This failure of mitochondrial adaptation in the diabetic rats was unexpected and led us to consider vascular mitochondrial function and turnover as new therapeutic targets in diabetes.

To target mitochondrial dysfunction, we have explored connections between known mitochondrial regulatory pathways and established vascular consequences of diabetes. Endothelial dysfunction is an established vascular abnormality in diabetes and it is known to be regulated, in part, by endothelial nitric oxide synthase (eNOS) (77). Work by Nisoli’s group established nitric oxide (NO) to be an upstream regulator of mitochondrial biogenesis in a variety of tissues (78). We therefore examined the effects that NOS inhibition might have specifically in vascular mitochondrial biogenesis. In a 2013 publication, we noted that inhibition of eNOS blocked mitochondrial adaptation to an exercise intervention in the aorta of Sprague Dawley rats (79)—affirming the important role NO plays in vascular mitochondrial biogenesis and the adaptive response to exercise.

## Pancreatic Dysfunction

Onset of exercise in individuals without diabetes is associated with decreased insulin secretion and increased glucagon secretion (80–82). Previous reports suggest that exercise is trophic for the pancreatic beta cell (83, 84). We are unaware of studies conducted to evaluate islet dysfunction and its interaction with exercise capacity. Pancreatic β cell dysfunction and failure are common culprits in the pathogenesis of diabetes. The process of aging can result in impaired carbohydrate metabolism, *via* both increased insulin resistance and impaired insulin secretion (85–89). Several factors have been shown to contribute to decreased insulin secretion in aging including reduced expression of β(2)-adrenergic receptors (90), decreased calcium signaling (91), and chronic oxidative stress (92). Consequently, it is plausible that aging in people with diabetes could be associated with abnormal ratios of glucagon to insulin with a subsequent impact on exercise capacity (93). It is also important to note the important role that pancreatic β cell mitochondria play in facilitating insulin secretion and that mitochondrial dysfunction in β cells is associated with beta cell failure and thus dysregulation of glucagon (94–96). This principle has been further substantiated recently in a study illustrating the deleterious effect of tacrolimus on β cell mitochondria and subsequent β cell failure (97). It is interesting to speculate that the mitochondrial impairment noted in aging and diabetes could deleteriously impact the metabolic flexibility needed for the mitochondria to adapt to an exercise challenge and further interfere with the acute adaptive response of the islet to an exercise bout. Evaluation of the islet response to exercise in people with uncomplicated diabetes is an area for future investigation.

## Targeting NOS Dysfunction

To determine if it was possible to counteract NOS inhibition and improve adaptive response to exercise training, we looked to the incretin class of insulin secretagogues: glucagon-like peptide 1 receptor agonists (GLP-1 RA) and dipeptidyl peptidase 4 inhibitors (DPP4). GLP-1 signals *via* G-protein-coupled receptors that are highly expressed in the vasculature and have been shown to stimulate eNOS and increase cyclic AMP (cAMP)—leading to enhanced endothelial function and tissue perfusion, plus improved muscle glucose utilization (98–101). To explore the effects of incretins on exercise, vascular function, and mitochondrial adaptation in diabetes, we first examined the effects of saxagliptin (a DPP4 inhibitor) in Goto-Kakazaki rats (a lean rat model of insulin resistant diabetes). Saxagliptin combined with exercise training stimulated eNOS and restored vascular mitochondrial expression (70).

In a more recent study, we treated human subjects with uncomplicated T2D with the GLP-1 RA exenatide (102). Treatment with exenatide, without a concomitant exercise intervention, led to improvement of both diastolic cardiac function and aortic stiffness, but did not improve VO_2_ max or endothelial dysfunction as measured by flow mediated dilatation (102). These data suggest that an effective stand-alone intervention (without concomitant exercise intervention) would need to impact cardiac, vascular, and muscle function (the latter was not evaluated in this trial). In our clinical study, we did not measure the impact of exenatide on eNOS or NO. It is possible that eNOS dysfunction, including eNOS uncoupling in diabetes, rendered our subjects resistant to cAMP regulation of NO, or that the combination of GLP-1 RA and exercise training is needed to achieve an adaptive response and change VO_2_ max. These observations suggest that NO may be crucial for exercise adaptation in the vasculature. We are currently exploring other methods to target NOS dysfunction in the context of diabetes.

Other agents with “insulin sensitizing” mechanisms have been examined as exercise mimetics in non-diabetic preclinical models and healthy subjects, including AMPK stimulation and PPAR delta ligands. In a 2008 study, administration of an orally active AMPK agonist, AICAR, for 4 weeks resulted in a 44% enhancement of running endurance in sedentary mice (103). In a 2017 study, sedentary mice administered a PPAR delta ligand were able to run ~100 min longer than control mice before exhaustion, a 70% increase (104). Studies in diabetic models employing these agents are either underway or not yet conducted.

Sodium glucose transporter type 2 (SGLT2) inhibitors are a relatively new class of diabetes medications with demonstrated cardiovascular morbidity and mortality benefit in humans (105, 106). SGLT2 inhibitors have recently been found to improve insulin sensitivity in the skeletal muscle of diabetic rats (107) and in *ex vivo* human epicardial adipose tissue (108). SGLT inhibition with phlorizin was shown by Li et al. to improve endothelial dysfunction by way of increasing NO levels in human umbilical vein endothelial cells (109). We are not aware of any published studies directly addressing the effect of SGLT2 inhibition on CRF, except a beneficial impact in the context of heart failure (105, 106, 110). In light of the recent reports demonstrating improvements in cardiac function, aortic stiffness, and renal perfusion, it is plausible that SGLT2 inhibition will counteract the pathophysiological factors contributing to exercise impairment in diabetes (111–116). Further studies evaluating the impact of SGLT2 inhibition on insulin sensitivity, NO, and functional exercise capacity in diabetes are warranted.

## Muscle Perfusion-Microvasculature

The role of NOS in vascular adaptation to sheer stress and exercise is accepted. Still, it is unclear what the consequences of impaired NOS function are on skeletal muscle and cardiac function. Importantly, it is controversial whether the skeletal muscle and heart NOS isoforms (eNOS and neuronal NOS, nNOS) contribute to exercise training adaptation or whether it is NOS mediated blood flow that contributes to the NOS-dependent adaptive response. McConnell has reported that skeletal muscle mitochondrial adaptation to exercise training is unaffected by deletion of either eNOS or nNOS (117). Another recent study indicates that alterations in muscle microvascular blood flow after an exercise bout are needed to garner the bout effect of exercise on insulin action (118). In a parallel line of investigation, Liu and Barrett report that insulin-mediated microvascular recruitment is essential for skeletal muscle insulin action and that this response requires NOS stimulation (119, 120). Taken together, these reports support a model wherein skeletal muscle insulin action involves coordination of blood flow-dependent insulin delivery to the skeletal muscle and intact skeletal muscle insulin signaling.

Similar to the relationship between muscle microvascular perfusion and insulin action, cardiac and skeletal muscle perfusion are critical for physiological function during a bout of exercise. Our previous work demonstrated decreased myocardial perfusion by sestamibi during exercise in people with T2D—consistent with abnormal tissue perfusion associated with cardiac dysfunction in T2D (19). Further, we have also reported slowed skeletal muscle blood flow at the onset of exercise in people with T2D (22). With regard to skeletal muscle mitochondrial dysfunction, a few key questions are currently unresolved: 1. What is the relationship between *in vivo* skeletal muscle oxidative capacity and VO_2_ max? 2. What is the contribution of muscle perfusion to decreased skeletal muscle oxidative capacity? 3. Is the adaptive response to either an exercise bout or exercise training in the skeletal muscle impacted by diabetes?

We postulated that skeletal muscle perfusion limitations due to either capillary rarefication or perfusion heterogeneity may contribute to the limitations observed in muscle oxidative phosphorylation in people with diabetes, and ultimately the associated CRF impairment. In support of this model, we recently published that people with diabetes have similar capillary recruitment with the onset of exercise as controls, yet have decreased oxyhemoglobin depletion during exercise compared with people without diabetes (121). We postulate that decreased skeletal muscle oxidative capacity is due to a combination of skeletal muscle mitochondrial dysfunction and muscle perfusion abnormalities including irregular blood flow distribution. Ongoing work in our laboratory is investigating the relationship between muscle O_2_ delivery and muscle mitochondrial function.

**Figure 1:**
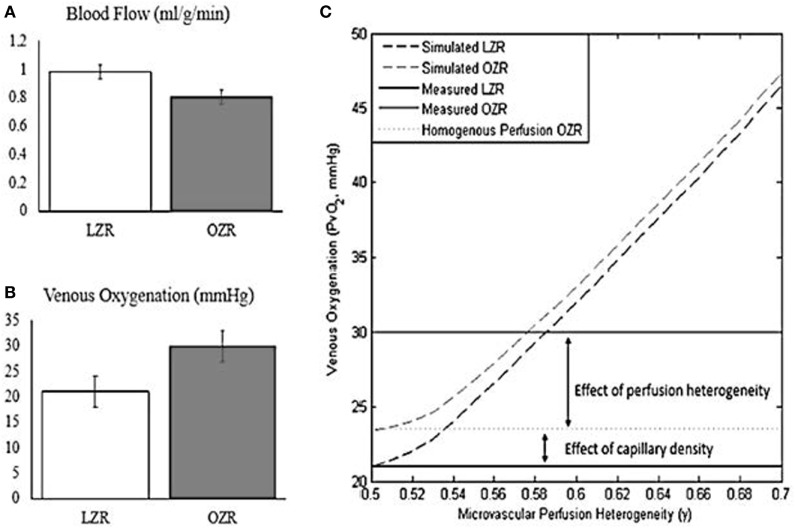
Oxygen extraction fails to compensate for reduced blood flow under conditions of microvascular perfusion heterogeneity. Adapted from Mason McClatchey et al. (123). Copyright 2017 Springer US. **(A)** Blood flow during exercise is reduced by ~20% in the obese Zucker rat (OZR) relative to the Lean Zucker Rat. **(B)** Venous oxygenation during exercise is increased in the OZR relative to the Lean Zucker Rat, reflecting impaired oxygen extraction. **(C)** Computational modeling of oxygen transport reveals that the failure to compensate for reduced blood flow in the OZR can be explained by the combined effects of reduced capillary density and microvascular perfusion heterogeneity.

To add to our understanding of lower effective muscle oxygen delivery in diabetes, we conducted a recent analysis of capillary density, muscle perfusion, and muscle fatigue in collaboration with Jefferson Frisbee’s group (122). This analysis combines a theoretical modeling simulation of blood flow distribution and predicted oxygen extraction across a muscle bed with experimental data from the lean Zucker rat and the metabolic syndrome obese Zucker rat (OZR) to test the contributors to decreased muscle VO_2_ in metabolic syndrome. Muscle blood flow was lower in the OZR and yet venous oxygen was higher. Simply stated, despite less blood flow delivery to the muscle there was less oxygen extracted to do the same amount of work (Figure 1). Possible explanations for this could be decreased capillary density or decreased effectively perfused capillaries. Based on our simulation, the observed diabetes-related decrease in capillary density only accounted for 20% of the mismatch. The remaining defect in muscle oxygen extraction was due to uneven blood flow (red blood cell) distribution. We were able to confirm that our modeling was correct by treating with an antioxidant (TEMPOL) and observing that we were able to restore ~80% of the mismatch (123). An acute response like this would not be possible if the defect was due to a fixed lesion such as decreased capillary density. Taken together, published findings indicate that the discrepancy in muscle oxygen extraction (muscle VO_2_) is primarily explained by muscle perfusion heterogeneity (122). In light of the significant improvements in oxygen extraction with the TEMPOL intervention, microvascular perfusion heterogeneity represents an unaddressed therapeutic target for improving tissue oxygenation and muscle function.

To summarize, muscle perfusion abnormalities are a plausible contributor to decreased VO_2_ and CRF in diabetes. Recent data in humans and in rodents using TEMPOL and other agents demonstrate that abnormalities in muscle perfusion can be corrected (123). Targeting muscle perfusion is a novel therapeutic direction for improving CRF and the adaptation to exercise intervention in diabetes. Future studies should focus on the impact of interventions targeting cardiac and skeletal muscle blood flow and examining the interaction between perfusion and the adaptive exercise training response.

## Cardiac Function

Studies to evaluate cardiac abnormalities using invasive and non-invasive methods revealed that cardiac abnormalities are present in newly diagnosed adults and youth with T2D upon exercise challenge (23, 24). We employed right heart catheterization to investigate cardiac function during exercise in sedentary, overweight premenopausal women with and without uncomplicated T2D. We found no differences in cardiac output at rest or during exercise (19). However, we did observe significantly increased PCWP during exercise in the T2D group suggesting the possibility of subclinical decreased ventricular contractility. This increase in PCWP correlated with decreased myocardial perfusion as assessed using sestamibi during stress testing (19). Similar differences were observed in adolescents with and without diabetes using non-invasive methods (23, 24). A clearer understanding of the long-term consequences of these subclinical CV changes is needed. In addition, it is unknown whether the agents that improve skeletal muscle perfusion will have the same impact on myocardial perfusion. Finally, the relationship between diastolic dysfunction, increased PCWP, and perceived rate of exertion may reveal an impact of cardiac dysfunction on CRF that is distinct from cardiac output. New MRI techniques may help elucidate changes and aid in evaluating the cardiac functional changes responsive to pharmacological or exercise interventions.

## Summary

People with even recently diagnosed diabetes have well-described aerobic exercise impairment as evidenced by decreased CRF with both maximal and submaximal workloads when compared with similarly overweight, sedentary non-diabetic subjects. Despite improvement in CRF after exercise training, exercise impairments persist in people with T2D compared with people without T2D for reasons that are not completely clear.

**Figure 2:**
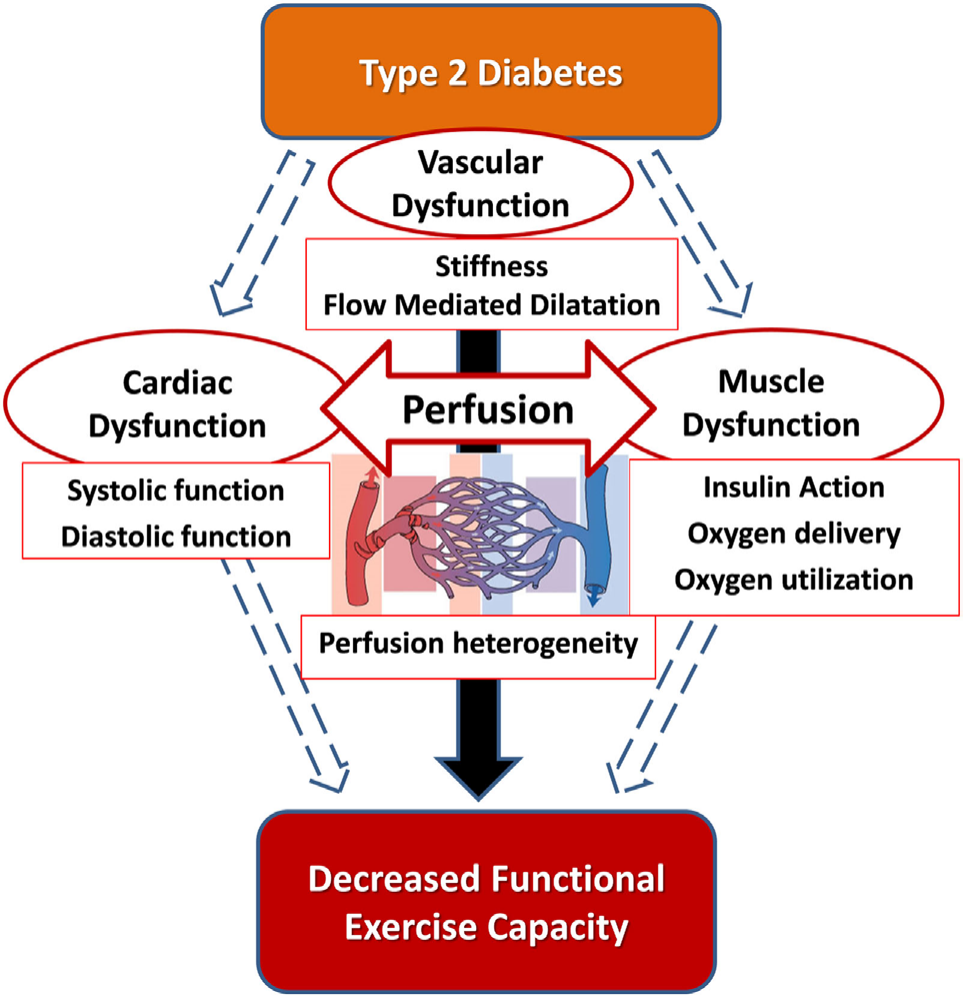
The hypothesis that the integration of cardiac function, macrovascular function, and microvascular function is impaired in type 2 diabetes and correlates with cardiorespiratory fitness impairment.

Diabetes is associated with exercise-induced abnormalities in the heart, skeletal muscle, and vasculature (Figure 2). Thus, the abnormalities are multifactorial which makes it unlikely that a single therapeutic approach will alleviate the entire problem. Therefore, approaches to resolve the exercise impairment in T2D should be multifactorial and target the contributors that have been identified across the cardiac, vascular, and skeletal muscle parameters in an integrative approach.

## Author Contributions

MW drafted the manuscript and revised it. RS edited and revised the manuscript. MW and RS developed the figures/tables. JGR and JEBR outlined and edited the manuscript and contributed to generation of data in the manuscript.

## Conflict of Interest Statement

The authors declare that the research was conducted in the absence of any commercial or financial relationships that could be construed as a potential conflict of interest.
